# Interaction of Nitrite Ions with Hydrated Portlandite Surfaces: Atomistic Computer Simulation Study

**DOI:** 10.3390/ma16145026

**Published:** 2023-07-16

**Authors:** Evgeny V. Tararushkin, Vasily V. Pisarev, Andrey G. Kalinichev

**Affiliations:** 1 International Laboratory for Supercomputer Atomistic Modelling and Multi-Scale Analysis, HSE University, 101000 Moscow, Russia; etararushkin@hse.ru (E.V.T.); vpisarev@hse.ru (V.V.P.); 2 Joint Institute for High Temperatures of the Russian Academy of Sciences, 125412 Moscow, Russia; 3 Laboratoire SUBATECH, UMR 6457–Institut Mines Télécom Atlantique, Nantes Université, CNRS/IN2P3, 44307 Nantes, France

**Keywords:** portlandite, hydration, nitrite, NO_2_^−^, adsorption, hydrogen bonding, molecular dynamics simulation

## Abstract

The nitrite admixtures in cement and concrete are used as corrosion inhibitors for steel reinforcement and also as anti-freezing agents. The characterization of the protective properties should account for the decrease in the concentration of free NO_2_^−^ ions in the pores of cement concretes due to their adsorption. Here we applied the classical molecular dynamics computer simulation approach to quantitatively study the molecular scale mechanisms of nitrite adsorption from NaNO_2_ aqueous solution on a portlandite surface. We used a new parameterization to model the hydrated NO_2_^−^ ions in combination with the recently upgraded ClayFF force field (ClayFF-MOH) for the structure of portlandite. The new NO_2_^−^ parameterization makes it possible to reproduce the properties of hydrated NO_2_^−^ ions in good agreement with experimental data. In addition, the ClayFF-MOH model improves the description of the portlandite structure by explicitly taking into account the bending of Ca-O-H angles in the crystal and on its surface. The simulations showed that despite the formation of a well-structured water layer on the portlandite (001) crystal surface, NO_2_^−^ ions can be strongly adsorbed. The nitrite adsorption is primarily due to the formation of hydrogen bonds between the structural hydroxyls on the portlandite surface and both the nitrogen and oxygen atoms of the NO_2_^−^ ions. Due to that, the ions do not form surface adsorption complexes with a single well-defined structure but can assume various local coordinations. However, in all cases, the adsorbed ions did not show significant surface diffusional mobility. Moreover, we demonstrated that the nitrite ions can be adsorbed both near the previously-adsorbed hydrated Na^+^ ions as surface ion pairs, but also separately from the cations.

## 1. Introduction

Cement-based concretes are capillary-porous materials that are often exposed to an aggressive environment (sulfate and chlorine attacks, alkali–aggregate expansion reactions, etc.). This can detrimentally affect the durability of structures made of concrete and reinforced concrete. To increase their durability and sustainability, various chemical additives are used. The application of such chemical additives is one of the effective solutions to green chemistry in concrete production [1]. Calcium and sodium nitrites, Ca(NO_2_)_2_ and NaNO_2_, are important chemical admixtures in the manufacture of concretes based on Portland cement. They inhibit the corrosion of reinforcing steel in reinforced concrete [2,3] and also act as anti-freezing agents [4,5]. It has been found that the nitrite ions, NO_2_^−^, bind to the cement surfaces [6]. This process reduces the concentration of free hydrated NO_2_^−^ in aqueous solutions inside concrete pores, thus reducing the effect of corrosion inhibition in the presence of Cl^−^ ions. Therefore, molecular scale understanding of the hydrated NO_2_^−^ behavior at the surfaces of cementitious materials is important for reliable prediction of the durability of reinforced concrete structures.

Portlandite, Ca(OH)_2_, is one of the important mineral phases of hydrated Portland cement, which is involved in the formation of the crystalline framework of hardened cement. The presence of portlandite maintains the high pH of the hardened cement [7], which is another factor protecting the reinforcing steel from corrosion [8]. In this paper, we chose this crystalline phase of hardened cement to quantify, by means of classical atomistic simulations, the structural, dynamic, and energetic aspects of its interactions with nitrite ions. According to experimental data [6], the use of the Ca(NO_2_)_2_ admixture can lead to the formation of new Ca(OH)_2_ crystals on the surface of existing portlandite crystals. This might introduce some undesirable ambiguity into the definition and quantitative analysis of the portlandite-solution interface within our current molecular modeling approaches. Therefore, NaNO_2_ rather than Ca(NO_2_)_2_ was selected for our simulations as a model nitrite admixture.

Methods of atomistic computer simulations make it possible to obtain detailed quantitative information about the adsorption and binding of ions on various surfaces at a fundamental molecular scale. In recent years, they have increasingly become an important and powerful tool in cement research [9,10,11]. The methods of atomistic computational modeling can be roughly subdivided into deterministic and stochastic. The stochastic approach of Monte Carlo (MC) simulations offers a statistical–mechanical description of materials, which is most useful for studying various thermodynamic characteristics, the energetics of adsorption, and other equilibrium properties of the simulated system under given thermodynamic conditions [12,13]. The deterministic approaches of molecular dynamics (MD) are based on the numerical solution of a set of Newtonian equations of motion for a large number of interacting particles (atoms, molecules, ions). In addition to thermodynamic properties, the MD approach also allows the calculation of many important dynamic properties, such as transport coefficients, vibrational spectra, or various relaxation times in the simulated system [14,15]. In the present work, we employed the method of classical MD simulations to model portlandite and its interaction with an interfacial aqueous solution of sodium nitrite. The recently modified version of the ClayFF force field (ClayFF-MOH) [16] with explicit Metal-O-H (MOH) angular bending terms [17] was used to simulate portlandite, while for the hydrated NO_2_^−^ ions a new parametrization of intermolecular interaction constants was recently developed [18]. Recent molecular simulations of the interfaces between aqueous salt solutions and another significant cement phase of ettringite [19] have already clearly demonstrated the importance of the MOH terms for accurate modeling of the mineral systems containing structural and interfacial hydroxyl groups. In addition to a more accurate reproduction of the properties of hydroxide minerals themselves, the introduction of MOH terms also affects the structural and dynamic properties of aqueous solutions in the near-surface layers of solutions compared to the original version of the ClayFF force field [20,21].

## 2. Models and Methods

### 2.1. Structural Models

Portlandite belongs to the group of metal hydroxides; M(OH)_2_ (M stands for a divalent metal cation), and has a brucite-like layered structure. Portlandite crystals have $P\bar{3}m1$ symmetry and the crystal structure consists of alternating trioctahedral layers along the *c*-axis that terminate with hydroxyl groups. The layers are formed by distorted edge-sharing CaO_6_ octahedra, with each hydroxyl ion being linked to three calcium ions [22,23].

An ideal unit cell of portlandite used in this work was built according to the structure determination by neutron diffraction measurements [24] with the lattice parameters of *a* = 3.59 Å, *c* = 4.90 Å, *γ* = 120°. A supercell of 10 × 10 × 8 unit cells along the *a*, *b*, and *c* cell vectors, respectively, was constructed for our simulations. The supercell had dimensions of approximately 35.9 × 35.9 × 39.3 Å^3^ and contained 4000 atoms.

To simulate the interface of portlandite with NaNO_2_ aqueous solution, a system consisting of 11 × 10 × 8 portlandite unit cells was cleaved parallel to the (001) plane and cut in the ab plane of the crystal to a rectangular shape. The (001) interface orientation is chosen because it has one of the lowest cleavage-free energies of all possible portlandite crystal faces [25,26,27,28]. Despite its hydrophilicity, the (001) crystal surface is more resistant to dissolution in water compared to other crystal faces of portlandite and brucite [29,30,31].

The created portlandite surfaces were brought into contact with a layer of NaNO_2_ aqueous solution approximately 85 Å thick. According to experimental data [32], this size is comparable to typical capillary pores in hardened cement. After the insertion of the aqueous layer, the dimensions of the simulation supercell were ~71.7 × 62.1 × 122.8 Å^3^. The number of H_2_O molecules in the nanopore corresponded to the density of the aqueous solution under ambient conditions (≈1 g/cm^3^). The Na^+^ and NO_2_^−^ ions were initially uniformly distributed in the nanopore. The resulting nominal NaNO_2_ solution molality in the pore space was ≈0.56 mol/kg (12,632 H_2_O molecules and 64 ion pairs). This corresponds to the experimentally measured solution molalities in the pores in cement materials [6].

**Figure 1:**
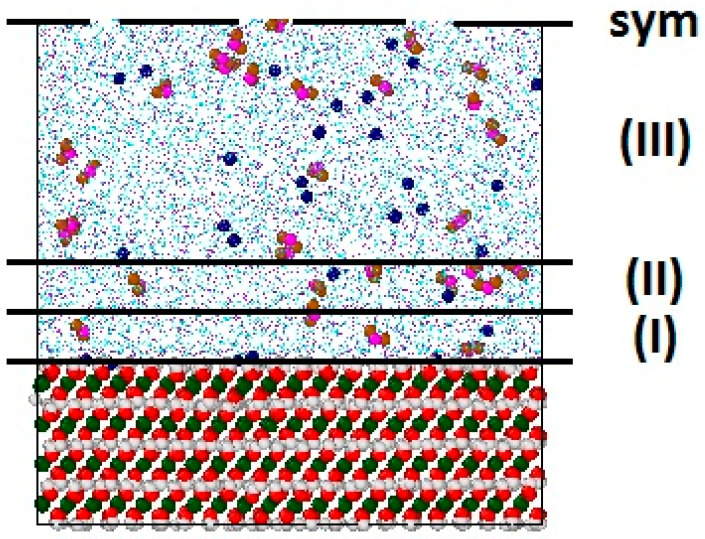
A representative snapshot of the simulation supercell of portlandite with NaNO_2_ aqueous solution. The thick black lines schematically indicate the first 3.5 Å (**I**), second 3.5 Å (**II**) solution layers next to the surface, and the bulk phase (**III**). **sym** is the symmetry plane of this slit-like pore.

Figure 1 shows a representative snapshot fragment of the simulation supercell. For the subsequent analysis, the interfacial space occupied by the solution was subdivided into three layers. The first near-surface layer (I) starts from the average positions of hydroxyl hydrogen atoms on the portlandite surface, H_h_, and has a thickness of 3.5 Å (approximately one molecular diameter of H_2_O. The second layer (II) immediately follows it and has the same thickness of 3.5 Å. In the rest of the pore space (III), the solution is considered to be much less affected by the surface and has properties close to those of the bulk phase.

### 2.2. Force Field Parameters

A series of preliminary simulations for the bulk Ca(OH)_2_ crystals was initially performed with both ClayFF-orig and ClayFF-MOH versions of the force field, in order to assess the relative accuracy of both versions. The MOH variation adds harmonic energy terms for Metal-O-H (M-O-H) angle bending in the crystal structure. The equilibrium angles and stiffness parameters were originally reported for Mg-O-H and Al-O-H angles [17]. They have been fitted to reproduce the lattice structure and dynamics obtained by density functional theory (DFT) calculations of brucite (Mg(OH)_2_)and gibbsite (Al(OH)_3_). The optimized parameters for Mg-O-H angles are as follows [16]: *θ*_0,MgOH_ = 110° and *k*_MgOH_ = 6 kcal∙mol^−1^∙rad^−2^. In this work, we use the same parameters for the Ca-O-H angles, based on the close similarity between the Mg(OH)_2_ and Ca(OH)_2_ crystal structures.

Water molecules were modeled using the flexible SPC/E model [33], and the parameters representing hydrated Na^+^ ions were taken from the updated ClayFF model [16]. As mentioned above, we used here a new parameterization of charges, Lennard-Jones constants, and intramolecular constants for the NO_2_^−^ ions in combination with the ClayFF force field [18]. This parameterization took into account recent results of DFT calculations for the nitrite ion dissolved in water [34,35], some earlier NO_2_^−^ models use in classical MD simulations [36,37,38], and experimental data [39]. All force field parameters used in this work were specifically parametrized to be consistent with the same SPC/E water model, therefore they are expected to be transferable and provide reliable results in the simulations of other systems that are consistent with the same model of water, as has been already demonstrated numerous times in the ClayFF-based simulations of various nanoporous and layered materials and their aqueous interfaces over the last 20 years [16].

### 2.3. Simulation Details

All MD simulations were performed using the LAMMPS simulation package [40]. LAMMPS is one of the most commonly used computer simulation software packages for modeling of cementitious materials and their interfaces with aqueous solutions (e.g., ref. [11]). Periodic boundary conditions in all three dimensions were used in all simulations. A cutoff radius of 12.5 Å was used for short-range forces, and the PPPM method was used to account for long-range electrostatic interactions [41,42]. The GPU accelerator within the LAMMPS package was used to speed up the calculations [42,43]. Standard Lorentz-Berthelot mixing rules ($\varepsilon_{ij}=\sqrt{\varepsilon_{ii}\varepsilon_{jj}};\;\sigma_{ij}=\left(\sigma_{ii}+\sigma_{jj}\right)/2$) were applied to calculate the Lennard-Jones parameters of interaction between unlike atoms [16,41]. The Newtonian equations of motion were numerically integrated with a time step of 0.5 fs via the velocity–Verlet algorithm [41,44].

The constructed models were initially equilibrated for 1 ns at the temperature of 298 K and pressure of 1 bar using the Nose-Hoover thermo-barostat [41,45,46]. After the equilibration in the *NPT* statistical ensemble (constant number of particles, constant temperature, and constant pressure), the equilibrium simulation run was performed in the *NVT* statistical ensemble (constant number of particles, constant temperature, and constant volume) using the Nose-Hoover thermostat [41,45,46]. The equilibrium *NVT* part was also 1.0 ns long and the generated equilibrium dynamic trajectories of atoms were then used for further statistical analysis.

The quantitative analysis of the structure, dynamics, and energetics of the portlandite-solution interfaces was performed following the standard approaches that are commonly used for such kinds of interfacial simulations (e.g., ref. [47]).

## 3. Results and Discussion

### 3.1. Bulk Portlandite Properties

#### 3.1.1. Crystallographic and Structural Parameters

Table 1 shows the equilibrium parameters of the crystallographic unit cell of portlandite calculated with the ClayFF-orig and ClayFF-MOH versions of the force field, in comparison with experimental data [24,48], DFT calculations [26,49,50], and earlier results of classical MD simulations [11,51,52,53,54]. In our simulations, all unit cell parameters were optimized in the NPT ensemble MD runs without any symmetry constraints.

The results clearly reproduced the hexagonal crystal structure, in agreement with experimental data, and the discrepancy of lattice parameters did not exceed 2.4% for the ClayFF-MOH version. Both versions of the ClayFF force field also showed a good agreement with the DFT results. There were no significant differences in the crystallographic parameters between the ClayFF-orig and ClayFF-MOH models, indicating that introducing the explicit M-O-H angle bending terms in portlandite does not affect the symmetry of the crystal. One has to note, however, that this agreement is not particularly surprising because portlandite was one of the model structures used to parametrize the original ClayFF force field [20,21].

**Figure 2:**
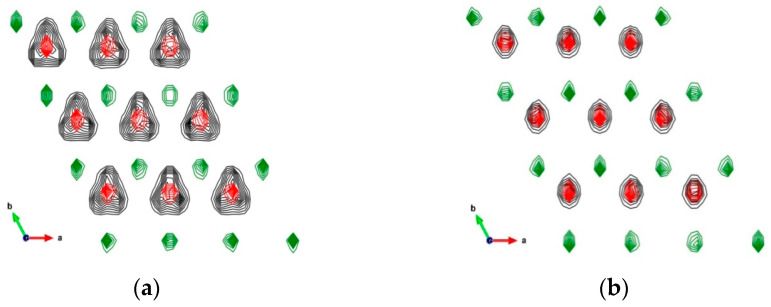
Time-averaged atomic density distributions of the layer of portlandite (supercell fragment from 3 × 3 unit cells). Green contours—Ca; red contours—O_h_; black contours—H_h_. (**a**) ClayFF-orig; (**b**) ClayFF-MOH.

The small differences in the crystallographic parameters between the ClayFF-orig and ClayFF-MOH models are due to the much stronger localization of the hydrogen atoms and the orientation of hydroxyl groups for the latter case (Figure 2). In the case of ClayFF-orig, the hydroxyl groups are freely oriented with their hydrogen atoms, H_h_, towards one of the three hydroxyl oxygen atoms, O_h_, of the opposite crystal layer, forming a strong hydrogen bond. Due to the unconstrained Ca-O-H angle in the ClayFF-orig, the hydroxyl groups can frequently and freely rotate, and those hydrogen bonds switch between three O_h_ atoms from the opposite layer. Such poorly constrained rotations of hydroxyl groups lead to a slight expansion of the interlayer space. Consequently, the parameter *c* of the portlandite unit cell increased, and the parameters *a* and *b* decreased compared to the ClayFF-MOH. As Figure 2a shows, the H_h_ atoms in the ClayFF-orig model can be oriented with equal probability in all three directions, corresponding to the locations of the O_h_ atoms in the neighboring layer (O_h_ atoms of the opposite layer are not shown).

**Figure 3:**
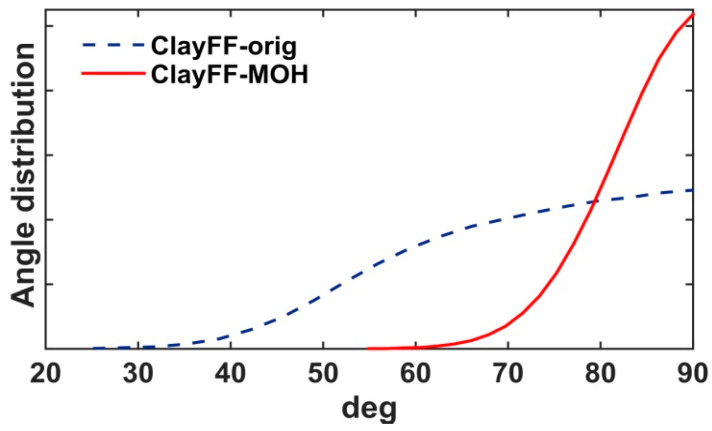
Hydroxyl slope angle density distributions.

Figure 2b demonstrates a much stronger localization of H_h_ atoms in the ClayFF-MOH model. Figure 3 shows the distributions of angles of hydroxyl groups to the *ab* plane in the original and ClayFF-MOH versions, which also reflect a more disordered behavior of hydroxyls in ClayFF-orig. In contrast, the hydroxyl O-H bonds are more strongly localized around the normal to the *ab* plane in the ClayFF-MOH model. According to the DFT results, such localized behavior of hydroxyls is more realistic of brucite-like materials [17] and is in better agreement with experimental data [22].

#### 3.1.2. Power Spectra of Atomic Vibrations in Portlandite Crystals

**Figure 4:**
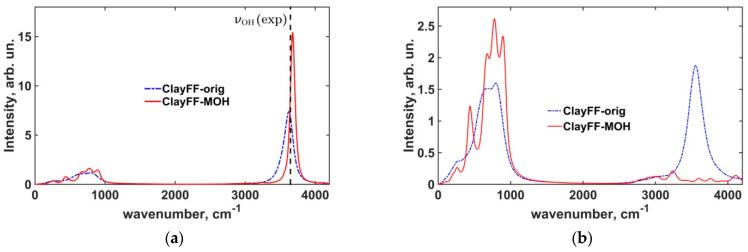
Power spectra of atomic vibrations. (**a**) total power spectra; (**b**) power spectra of H_h_-atoms in the *ab* crystallographic plane. (exp)—see [56,57].

Analysis of atomic vibration spectra in the crystal gives further insights into the dynamics of portlandite hydroxyls. Figure 4 shows smoothed power spectra reflecting the vibrations in the ab crystallographic plane for all portlandite atoms and, separately, the contribution of H_h_-atoms calculated in both versions of ClayFF. The smoothing of the spectra was carried out by applying the exponential damping with the characteristic time τ_damp_ = 0.25 ps to the velocity autocorrelation functions, as proposed by Szczerba et al. [55].

The low-frequency region of the spectrum corresponds to the librational modes of the hydroxyl groups and to the lattice vibrations. In the ClayFF-orig model, the individual peaks were unresolved and all merged into one single wide spectral band. In the ClayFF-MOH model, the individual peaks were better resolved for both libration and lattice vibration modes, which is in a better qualitative agreement with the vibrational behavior of portlandite crystal observed in experiments [56] and DFT calculations [50], compared to the ClayFF-orig model.

The most noticeable differences between the two versions of ClayFF in Figure 4a,b are in the high-frequency range of the spectra. The peaks corresponding to the O–H stretching modes of hydroxyls are located at positions 3624 cm^–1^ and 3676 cm^–1^ for the ClayFF-orig and ClayFF-MOH models, respectively. For the ClayFF-MOH, the peak frequency is slightly higher than the experimental data [56,57,58], where the O–H stretching peaks are located at 3620 cm^–1^ (A_1g_ mode) and 3646 cm^–1^ (A_2u_ mode) for Raman and IR spectroscopy, respectively. The lower frequency of the O–H stretching peak in the ClayFF-orig compared to the ClayFF-MOH indicates that the interlayer hydrogen bonding is stronger in the former model. As mentioned above, in the ClayFF-orig, H_h_ atoms instantaneously bond with only one opposite-layer O_h_ atom. In ClayFF-MOH, an H_h_ atom can participate in up to 3 weaker hydrogen bonds at the same time, due to the higher localization of H_h_ atoms in the structure. For instance, in brucite under normal conditions, the preferred orientation of the hydroxyl groups is along the normal to the magnesium octahedral layer [17,59]. The reorientation of hydroxyl groups in brucite and portlandite towards the opposite-layer O_h_ atoms occurs only at high temperatures and pressures [23,60]. This exaggerated reorientation of hydroxyls under normal conditions in the ClayFF-orig model is especially noticeable in the isolated spectrum of H_h_-atoms in the ab crystallographic plane, where a very strong peak is observed in the high-frequency region, as seen in Figure 4b.

### 3.2. Properties of Portlandite-NaNO_2_ Aqueous Solution Interfaces

#### 3.2.1. Structural Properties of Interfacial Solutions

**Figure 5:**
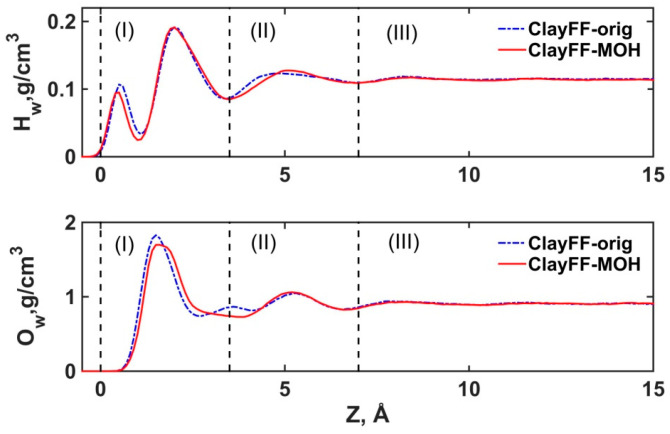
Atomic density distributions of H_w_ and O_w_ atoms for both versions of ClayFF force fields. (**I**) and (**II**) are the first and second solution layers, respectively. (**III**) denotes the bulk phase of the solution (see Figure 1).

First, we discuss the structural differences in the simulated aqueous solution between the two ClayFF versions. Figure 5 shows atomic density profiles of H_w_ and O_w_ atoms of the H_2_O molecules along the direction normal to the portlandite surface for both versions of ClayFF. As expected for the ClayFF-orig version, the profiles for H_w_ and O_w_ were similar to the earlier MD simulation results with this force field [20,51]. However, slightly different profiles are observed with the ClayFF-MOH model, indicating some changes in the local interfacial solution structure. Below, we argue that the reason for the changes in the density profiles in the first two solution layers, (I) and (II) (as defined in Figure 1) are directly related to very specific interfacial molecular coordinations.

DFT and MD results show that one of the most probable orientations of the H_2_O molecule dipole vector is 170–175° with respect to the surface normal [26,51]. With this orientation, the H_2_O molecule donates two hydrogen bonds to the O_h_ atoms of the hydroxyls on the surface of the crystalline phase and this orientation of the H_2_O molecule corresponds to the bidentate coordination towards the surface [55]. In addition, there may be a monodentate coordination with the dipole vector oriented ≈90° to the surface normal [55]. Such orientations of H_2_O molecules on the (001) surface of portlandite have been reported in other recent MD simulations [61]. Due to the more accurately constrained vertical orientations of the surface hydroxyl groups with the ClayFF-MOH model, H_2_O molecules in those two orientations are adsorbed closer to the portlandite surface. That, in turn, results in stronger hydrogen bonds in the donor-acceptor pairs H_w_···O_h_ and H_h_···O_w_. The stronger bonding of H_2_O molecules in mono- and bidentate orientations is reflected in the shift of the first peak of the H_w_ profile closer to the portlandite surface (in layer (I)) compared to the ClayFF-orig version.

However, the O_w_ and H_w_ interfacial density profiles for both versions of the force field clearly reproduce the distinct layering of water at least within the first two molecular layers, which is fully consistent with the results of previous simulations [20,51,61] and justifies our definition of the solution layers in Figure 1.

The slight shift of the first minimum of the H_w_ profile in layer (I) for ClayFF-MOH (Figure 5) is also explained by the adsorption of the H_2_O molecules closer to the portlandite surface. The second peaks of the density profiles in layer (I) are located at the same positions for both versions of the ClayFF model. These peaks are the highest and correspond to the most common orientation of H_2_O molecules, in which interfacial H_2_O molecules accept hydrogen bonds from surface hydroxyls (donor-acceptor pair H_h_···O_w_) and H_2_O molecules are located along the hydroxyl O-H bonds. In the second layer (II), the H_w_ density maximum shifts away from the surface for the ClayFF-MOH model compared to ClayFF-orig. The reason for this is that the mono- and bidentate-oriented H_2_O molecules in layer (I) are coordinating the H_2_O molecules from layer (II) in a slightly different manner due to more stable hydrogen bonds with the surface observed with the ClayFF-MOH model. These changes are reflected in the O_w_ profiles as well, where there are two peaks In the near-surface layer (I) and (II) for the ClayFF-MOH compared to three peaks for the ClayFF-orig. The broadening of the first peak and the absence of a peak at the boundary of layers (I) and (II) In ClayFF-MOH indicate the formation of a stronger hydrogen bonding network on the surface of portlandite. In general, the H_2_O atomic density profiles for the ClayFF-MOH are qualitatively more similar to the H_2_O profiles from the MD simulations with a more optimized force field for solid and a four-point TIP4P/2005 water model [61].

Thus, the ClayFF-MOH model provides a better qualitative and quantitative description of the portlandite crystal and the portlandite-water interface. Therefore, in the rest of the paper, the behavior of dissolved ions and H_2_O molecules on the portlandite surface are presented and discussed only for the ClayFF-MOH model.

**Figure 6:**
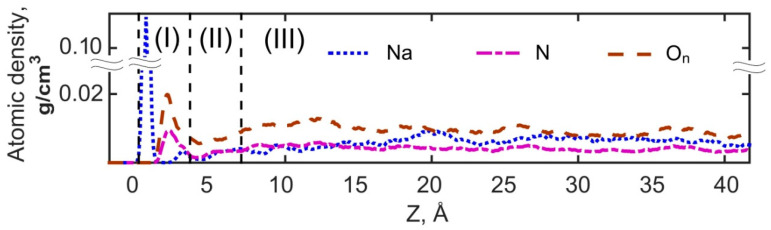
Atomic density distributions of Na^+^ and NO_2_^−^. Half of the nanopore is shown due to the symmetry of the model. (**I**) and (**II**) are the first and second layers of the solution, respectively. (**III**) denotes the bulk phase of the solution (see Figure 1).

Figure 6 shows the density profiles of Na^+^ ions and N and O_n_ atoms of NO_2_^−^ in the sodium nitrite aqueous solution next to the portlandite surface. The density profile of Na^+^ is similar to the previously reported profiles [20,51] with Na^+^ is mainly adsorbed with inner-sphere surface coordination. Inner-sphere coordination is also observed for adsorbed NO_2_^−^ ions, as the first density peaks of N and O_n_ are located within layer (I) of the solution at distances of 2.1 and 2.0 Å from H_h_, respectively. The first minima of the profiles are located in both cases within the layer (II) of solution at distances of 4.0 Å for N and 4.1 Å for O_n_, indicating that adsorbed NO_2_^−^ ions can be oriented both with their N and O_n_ atoms towards the H_h_ hydroxyls. In both orientations NO_2_^−^ ions accept hydrogen bonds from surface hydroxyls.

**Figure 7:**
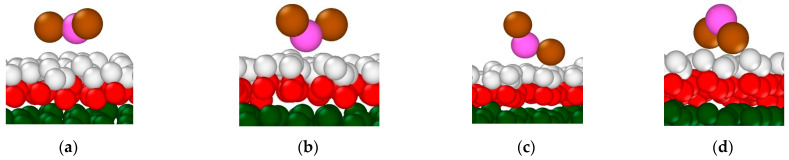
NO_2_^−^ ion coordination on the portlandite surface (explanation for subfigures **a**–**d** please see Section 3.2.1 for a detailed explanation and discussion). Green sphere—Ca; red sphere—O_h_; white sphere—H_h_; magenta sphere—N; brown sphere—O_n_.

Visual analysis of the trajectories with the OVITO package [62] has shown that the adsorbed NO_2_^−^ ions demonstrate four main types of surface coordination as illustrated in Figure 7a–d. In the first coordination (Figure 7a), all three atoms of the NO_2_^−^ ion are in a plane oriented almost parallel to the portlandite surface and coordinated by surface hydroxyls. The other three orientations have only the N atom, one O_n_ atom, or both O_n_ atoms coordinated by the surface hydroxyls, as shown in Figure 7b, Figure 7c and Figure 7d, respectively. These types of coordination can be seen for the anions adsorbed next to Na^+^ ions on the surface as well as for the anions located far from adsorbed cations. As mentioned above, the first minima of the N and O_n_ density profiles are located within the solution layer (II), so that the density of NO_2_^−^ in that layer is lower than in layer (I) and in layer (III) that reflects bulk solution properties. This behavior of NO_2_^−^ in layer (II) is caused by the repulsion from the first NO_2_^−^ adsorption layer in layer (I).

**Figure 8:**
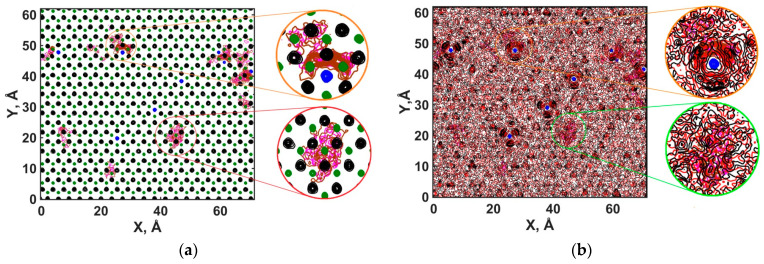
Contour maps of atomic density for solution species at the surface of portlandite (solution layer (I)). Green contours—Ca (**a**); black contours—H_h_ (**a**) and H_w_ (**b**); red contours—O_w_ (**b**); blue contours—Na^+^ (**a**,**b**); purple contours—N (**a**,**b**); brown contours—O_n_ (**a**,**b**). (**a**) Crystalline phase and ions of an aqueous solution; (**b**) ions of an aqueous solution and atoms of H_2_O molecules.

The surface distribution of molecules visualized by atomic density contour maps for the solution layer (I) shows that Na^+^ ions are adsorbed at the centers of triangles formed by O_h_ atoms, as illustrated in Figure 8. The inner-sphere adsorption of Na^+^ ions and the coordination of the H_2_O molecules around them by O_w_ atoms agrees well with the previous MD results [20,51]. Unlike Hu et al. [63], we did not observe the preference of NO_2_^−^ adsorption surrounding the cations and forming surface clusters. Instead, NO_2_^−^ ions can be adsorbed both near Na^+^ and far from them. In both cases, the adsorbed anions are quite strongly bound to the surface and do not change their binding site within the time scale of the simulation. Nevertheless, the nitrite anions frequently change their orientation while staying at the same surface adsorption site. Due to the frequent changes of adsorbed NO_2_^−^ ions orientation, the H_2_O molecules that form hydrogen bonds with these NO_2_^−^ ions did not have any predominant coordination type, as seen in Figure 8b.

#### 3.2.2. Power Spectra of the Interfacial Dynamics

**Figure 9:**
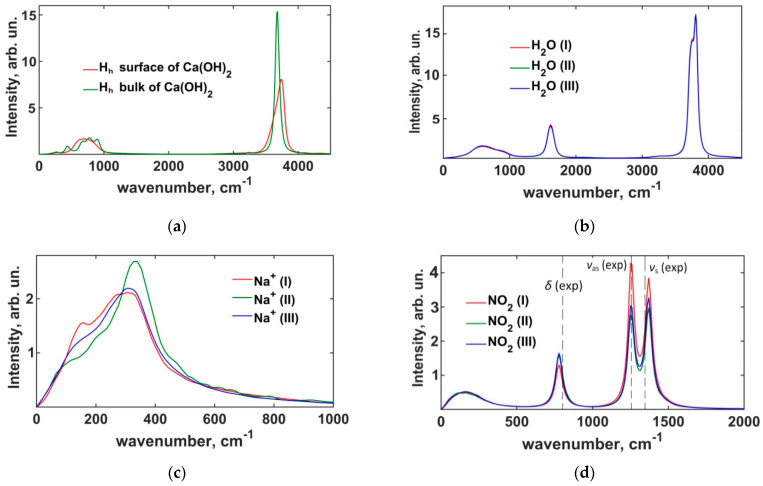
Power spectra of atomic vibrations for crystalline phase and ionic species and H_2_O molecules: (**a**) H_h_; (**b**) H_2_O; (**c**) Na^+^; (**d**) NO_2_^−^. (exp)—see [39]. (**I**) and (**II**) are the first and second layers of the solution, respectively. (**III**) denotes the bulk phase of the solution (see Figure 1).

Power spectra reflecting the vibrations of H_h_ atoms at the surface and in the bulk structure of portlandite are shown in Figure 9a. As mentioned in Section 3.1.2, the low-frequency region of the bulk portlandite spectrum has a band where several peaks can be resolved. In contrast to that, only two peaks can be resolved in that band for surface H_h_, which indicates a superposition of normal vibration modes for surface hydroxyls. In the high-frequency range, the peak corresponding to the stretching of O–H bonds is broader, less intense, and significantly blue-shifted (3676 cm^–1^ vs. 3743 cm^–1^) for surface hydroxyls compared to the bulk phase, which is typical for surfaces of brucite-like minerals [59]. The shift of the O–H stretching vibrations peak for surface H_h_ at the crystal–solution interface is significantly smaller than with the crystal-vacuum interface [59]. The reduced peak shift is due to the formation of hydrogen bonds of the surface hydroxyls with the Interfacial H_2_O molecules and NO_2_^−^ ions. Figure 9b shows that the power spectra of the H_2_O solution molecules at all distances from the surface are identical. This may merely reflect the fact that simple harmonic potentials used here to model the intramolecular vibrations in the SPC/E water model are not accurate enough, and more complex inharmonic models are necessary (see, e.g., ref. [55]). However, the libration, bending, and stretching modes of H_2_O molecules are reproduced with only small deviations from the experimental data [64,65].

Unlike the case with H_2_O molecules, the spectra reflecting the surface dynamics of Na^+^ ions show some dependence on the distance from the surface, as seen in Figure 9c, which confirms the previous MD results [20], where such spectra have been analyzed in detail. However, the NO_2_^−^ power spectra in different solution layers have peaks at the same frequencies, differing only in magnitudes, as shown in Figure 9d. The positions of the spectral peaks are in good agreement with the results of Raman spectroscopy [39]: the calculated frequency of the bending mode, *δ*, is at 779 cm^–1^, compared to 817 cm^–1^ from the Raman spectroscopy. The calculated asymmetric, *ν*_as_, and symmetric, *ν*_s_, N-O_n_ stretching modes peaks are at 1250 cm^–1^ and 1371 cm^–1^, respectively, compared to the experimentally determined values of 1242 cm^–1^ and 1331 cm^–1^.

#### 3.2.3. Structure and Dynamics of the Interfacial Hydrogen Bonding

**Figure 10:**
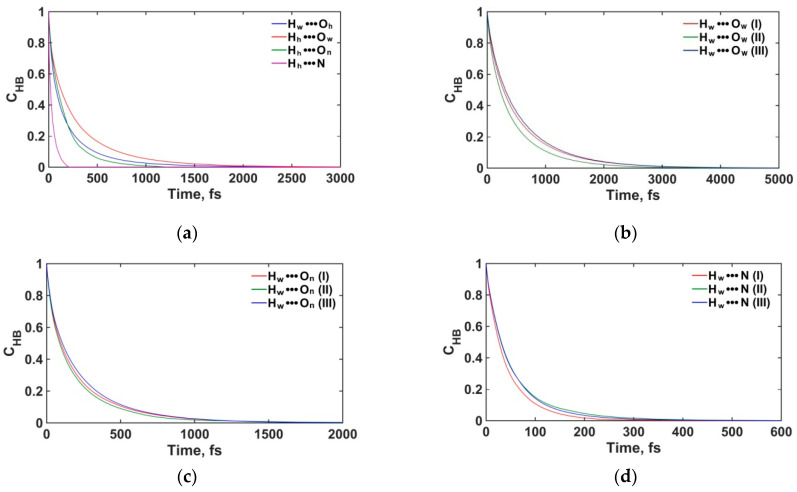
Continuous time autocorrelation functions. (**a**) H_w_···O_h_, H_h_···O_w_, H_h_···O_n_, H_h_···N; (**b**) H_w_···O_w_; (**c**) H_w_···O_n_; (**d**) H_w_···N. (**I**) and (**II**) are the first and second layers of the solution, respectively. (**III**) denotes the bulk phase of the solution (see Figure 1).

To better characterize the structural and dynamic properties of the interfacial solution, we have also studied the dynamics of hydrogen bonds (H-bonds), which were formally defined using a common geometric criterion: an H-bond between a hydrogen H_w_ (donor) and oxygen O_w_ (acceptor) atoms of two H_2_O molecules was assumed to exist if the H_w_···O_w_ distance is less than 2.45 Å and the angle between the H-bond direction and the vector connecting the O_w_ atoms of the donor and acceptor molecules is less than 30° (e.g., ref. [66]). For H-bonds between H_2_O molecules and NO_2_^−^ (H_w_···N and H_w_···O_n_ pairs), the distance thresholds were taken according to [35]: R_Hw-N_ ≤ 2.25 Å and *R*_Hw-On_ ≤ 2.35 Å. The geometrical definitions for H-bonds between portlandite surface hydroxyls and H_2_O (or NO_2_^−^) were taken to be the same as for H_2_O molecules (or H_2_O and NO_2_^−^) in solution. The average lifetimes *τ*_HB_ for various types of H-bonds were then assessed by integrating the so-called continuous-time autocorrelation functions [35,66,67,68] as illustrated in Figure 10. In addition, the average number of hydrogen bonds *n*_HB_ per H_2_O molecule and acceptors N and O_n_ were also calculated.

It was found that the portlandite hydroxyls form the most stable H-bonds with the H_2_O molecules of the solution (pairs H_h_···O_w_ and H_w_···O_h_). The lifetime of the H_h_···O_w_ bonds is longer than that for H_w_···O_h_, despite the fact that in the ClayFF model, the charge of O_h_ is more negative than that of O_w_, while the charges of H_h_ atoms and H_w_ atoms in SPC/E are almost equal. In the bidentate orientation of H_2_O molecules, which is most preferable on the surface of portlandite [26], H_2_O molecules are also coordinated by neighboring hydroxyls (H_h_···O_w_ pairs) without forming hydrogen bonds between H_w_ and O_h_ of this hydroxyls.

As mentioned above, monodentate orientation can also occur, in which H-bonds between the H_h_···O_w_ and H_w_···O_h_ pairs are formed. Therefore, a significant role on the hydrated (001) portlandite surface is played by the H-bonds donated by the hydrogen atoms of the surface hydroxyl groups to the oxygen atoms of H_2_O molecules (pair H_h_···O_w_), which inhibits the penetration of H_2_O molecules closer to the portlandite surface and the dissolution of calcium ions [29].

Table 2 shows that the lifetime of H-bonds between H_2_O molecules in the solution layer (I) is longer than in the layer (II), while the number of H-bonds per H_2_O molecule remains about the same for both near-surface layers. This means that the interaction of H_2_O molecules with surface hydroxyls orients them in a way that favors the formation of a more stable H-bond network between themselves in layer (I). That H-bonding network of the H_2_O molecules nearest to the surface (in layer (I)) creates an ice-like two-dimensional layer on the surface of portlandite [29]. In the bulk solution (layer (III)), the number of H-bonds per H_2_O molecule and the bond lifetime have typical values for low-concentration aqueous solutions [69].

For interactions between water molecules and nitrite ions, the H-bonds H_w_···O_n_ are stronger than H_w_···N, which is consistent with the DFT results for hydrated NO_2_^−^ [35]. The H-bonds for these pairs have approximately the same lifetimes in all layers of the aqueous solution. However, the average number of H-bonds per acceptor atom in H_w_···N pairs in layer (I) is lower than in layer (II), because some of the H_2_O molecules in layer (I) bind with surface hydroxyls instead of NO_2_^−^ nitrogen atoms. The lifetime of H_h_···N bonds between hydroxyls and NO_2_^−^ is close to the lifetime of the H_w_···N bond, however, the H_h_···O_n_ bonds have shorter lifetimes than Hw···On. Such a decrease in the lifetime is due to the vibrational behavior of the surface hydroxyls of the crystalline phase, which is also noticeable when comparing the lifetimes of the H_h_···O_w_ and H_w_···O_w_ pairs. We can also see that the H-bonds between surface hydroxyls and adsorbed NO_2_^−^ ions are weaker than the ones between hydroxyls and H_2_O molecules. This is consistent with the absence of a strongly preferred NO_2_^−^ orientation on the portlandite surface.

#### 3.2.4. Water Molecules and Nitrite Ions Orientational Relaxation

**Figure 11:**
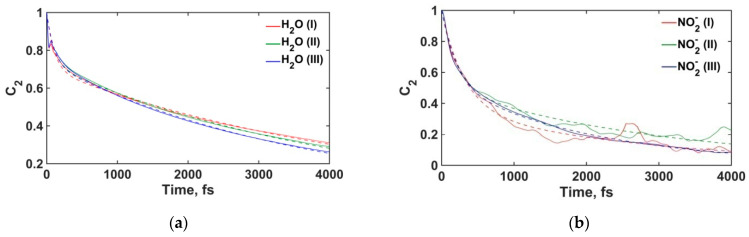
Time correlation functions. Dashed lines represent the biexponential fits. (**a**) H_2_O molecules; (**b**) NO_2_^−^ ions. (**I**) and (**II**) are the first and second layers of the solution, respectively. (**III**) denotes the bulk phase of the solution (see Figure 1).

The orientational relaxation of NO_2_^−^ ions and H_2_O molecules can be characterized using the autocorrelation function of the unit vectors directed along the N-O_n_ or O_w_-H_w_ bonds [35,70,71,72], which was calculated using the second-rank Legendre polynomial:

(1) $$C_{2}\left(\cos\theta \right)=\langle \frac{3}{2}\cos^{2}\theta -\frac{1}{2}\rangle ,$$

where $\cos\theta \left(t\right)=d^{T}\left(t_{0}\right)d\left(t_{0}+t\right)$ is the linear correlation between the unit vector of bond orientation d at time moments $t_{0}$ and $t_{0}+t$, and angular brackets 〈…〉 denote the averaging over all molecules and over all possible initial moments $t_{0}$ along the equilibrium MD trajectory. The correlation functions of NO_2_^−^ and H_2_O molecules in three layers of the aqueous solution are shown in Figure 11. The orientational correlation times were calculated by fitting *C*_2_ with bi-exponential functions [35]:

(2) $$C_{2}\left(t\right)=A_{1}\exp\left(-\frac{t}{\tau_{1}}\right)+A_{2}\left(-\frac{t}{\tau_{2}}\right),$$

where the shorter time constant *τ*_1_ reflects the rotational inertia of an ion or molecule in the libration mode, which is affected by H-bonding around the ion/molecule [71]. The longer time constant *τ*_2_ characterizes the reorientation of an ion or molecule due to the rotational Brownian motion and to large angular jumps associated with the breaking of H-bonds [35,71].

Table 3 shows the orientational relaxation times for NO_2_^−^ and H_2_O, depending on the molecule location in the pore. For H_2_O molecules, the time *τ*_1_ for all layers of the solution have similar values, indicating low sensitivity of librational molecular motions to the variation of local H-bonding environment between bulk solution and near-surface layers. The power spectra in Figure 9b also indicate the same behavior in the low-frequency region. On the other hand, the time *τ*_2_ for H_2_O molecules increases in the near-surface layers. Such an increase in rotational relaxation time confirms the aforementioned formation of a more stable H-bonding network at the surface of crystalline portlandite due to hydroxyl group–water interactions.

The increase in *τ*_2_ near the surface compared to their bulk solution values is seen for NO_2_^−^ ions as well, which is also due to the participation of the nitrite ions in the more stable H-bonding network at the surface of portlandite. In the closest near-surface layer, we also observe an increase in time *τ*_1_, indicating that the NO_2_^−^ ion adsorption affects its libration modes.

#### 3.2.5. Diffusional Mobility of Water and Ions at the Portlandite Surface

The translational dynamics of NO_2_^−^, Na^+^, and H_2_O molecules in the near-surface layers and in the bulk of the solution were studied by calculating the self-diffusion coefficients of these species using the Einstein–Smoluchowski relation [41,72,73]. For H_2_O molecules, we calculated the 3-dimensional (3d) diffusion coefficients separately for the solution layers (I), (II), and (III), as defined in Figure 1. For the ions, due to their relatively small number in the simulated systems, only bulk (layer (III)) 3-dimensional diffusion coefficients were calculated.

Table 4 shows the calculated self-diffusion coefficients. The average self-diffusion coefficients for the ions are found to be $D_{Na}$ = (0.84 ± 0.17) × 10^–5^ cm^2^/s, $D_{NO_{2}}$ = (1.46 ± 0.16) × 10^–5^ cm^2^/s. The calculated diffusivities of ions in water are lower than the experimental values $D_{Na}^{exp}$ = 1.33 × 10^–5^ cm^2^/s and $D_{NO_{2}}^{exp}$ = 1.91 × 10^–5^ cm^2^/s [74].

The diffusional mobility of water molecules decreases as they approach the surface, with $D_{H_{2}O}^{III}$ = (1.81 ± 0.16) × 10^–5^ cm^2^/s, $D_{H_{2}O}^{II}$ = (1.47 ± 0.18) × 10^–5^ cm^2^/s, and $D_{H_{2}O}^{I}$ = (1.36 ± 0.21) × 10^–5^ cm^2^/s. The diffusivity of water in the bulk layer is underestimated compared to the experimental data $D_{H_{2}O}^{exp}$ = 2.33 × 10^–5^ cm^2^/s [75]. The reduced calculated diffusivities of water and ions reflect the effect of their interactions with the portlandite surface but are also partly due to the known underestimation of the molecular mobility by the flexible SPC/E model and due to the finite size effects of the periodic boundary conditions [76].

## 4. Conclusions

The recently modified ClayFF force field, ClayFF-MOH [10], with added Ca-O-H angular bending terms, together with a new parameterization for NO_2_^−^ ions [18] were used to study the structure and dynamics of sodium nitrite solutions at the surface of portlandite using classical MD simulations. The (001) crystal surface of portlandite was selected for these simulations because it is the least reactive when interacting with water compared to other surfaces. We show that the inclusion of the Ca-O-H bending term does not affect the simulation results for the crystal structure but does affect the vibrational properties of the crystal, bringing them closer to the experimental data. This, in turn, affects the structural properties of the aqueous solutions in the near-surface layers due to the increased localization of surface hydroxyl groups of portlandite.

The better-constrained orientation of the hydroxyls on the (001) surface of portlandite leads to a more realistic description of the hydrogen bonds formed between surface hydrogen atoms and water molecules (H_h_···O_w_ donor-acceptor pair), facilitating the formation of a network of H-bonds between water molecules themselves, which form a surface layer inhibiting the dissolution of calcium ions into water. The formation of the interfacial H-bonding network manifests itself in the increase in the rotational relaxation time for water molecules near the portlandite surface.

Despite the formation of the water-water H-bonding network on the surface of the crystalline phase, the surface can adsorb dissolved nitrite and sodium ions. We show that the adsorption of nitrite ions involves the formation of H-bonds donated by surface hydroxyls not only to the oxygen atoms of NO_2_^−^, but also to the nitrogen atoms, i.e., both H_h_···O_n_ and H_h_···N donor-acceptor pairs are present. Although the H-bonds H_h_···O_n_ and H_h_···N are weaker than the H-bonds H_h_···O_w_ donated to the water molecules, NO_2_^−^ ions infiltrate the H-bonding network on the surface of portlandite and are quite strongly adsorbed at it with no visible translational diffusion over the timescale of the entire MD simulation (~1 ns). On the other hand, the orientation of the adsorbed ions can change over time, so that there is no single preferred coordination of the adsorbed NO_2_^−^ to the surface, meaning that the H-bonds in the H_h_···O_n_ and H_h_···N pairs are less stable and can frequently break and re-form. The NO_2_^−^ ions can be adsorbed onto the surface of portlandite next to the previously adsorbed Na^+^ ions as well as separately without any strong association with the cations.

## Figures and Tables

**Table 1:** Crystallographic unit cell parameters of portlandite: simulation results and experimental data.

Source	*a*, Å	*b*, Å	*c*, Å	*α*, °	*β*, °	*γ*, °
ClayFF-orig (This work)	3.6343	3.6357	4.8957	90.05	90.00	119.99
ClayFF-MOH (This work)	3.6779	3.6783	4.8767	90.03	89.98	119.99
ClayFF [51]	3.567	3.567	4.908	90	90	120
IFF [11]	3.75	3.75	4.38	90	90	120
CementFF [11]	3.68	3.67	4.81	90	90	120
CSH-FF [52]	3.5037	3.5037	4.8045	90	90	120
ERICA FF [53]	3.67 (2.2)	3.67 (2.2)	4.85 (0.4)	89.7	89.9	120
ReaxFF [54]	3.63	3.63	5.10	89.67	90.05	120.11
DFT [49]	3.609	3.609	4.864	90	90	120
DFT [26]	3.696	3.696	5.147	90	90	120
DFT [50]	3.581–3.625	3.581–3.625	4.797–5.010	90	90	120
Neutron diffraction [24]	3.5918	3.5918	4.9063	90	90	120
NMR [48]	3.5925	3.5925	4.905	90	90	120

**Table 2:** The average number of H-bonds per H_2_O molecule/acceptor (*n*_HB_) and the average lifetime of H-bond (*τ*_HB_).

Donor-Acceptor Pair	Layer	*n*_HB_	*τ*_HB_, ps
H_w_···O_w_	(I)	2.26	0.51
	(II)	2.23	0.41
	(III)	3.53	0.54
H_w_···O_n_	(I)	1.68	0.20
	(II)	1.67	0.18
	(III)	2.69	0.21
H_w_···N	(I)	0.89	0.04
	(II)	1.06	0.05
	(III)	1.64	0.05
H_w_···O_h_	(I)	0.27	0.18
H_h_···O_w_	(I)	0.43	0.27
H_h_···O_n_	(I)	0.45	0.16
H_h_···N	(I)	0.20	0.04

**Table 3:** The orientational relaxation times for H_2_O molecules and NO_2_^−^.

Molecule/Ion	Layer	*τ*_1_, ps	*τ*_2_, ps
H_2_O	(I)	0.11	4.76
	(II)	0.11	4.21
	(III)	0.10	3.78
NO_2_^−^	(I)	0.32	2.89
	(II)	0.22	3.11
	(III)	0.20	2.08

**Table 4:** Self-diffusion coefficients of ions and H_2_O molecules.

Molecule/Ion	Layer	2d, 10^−5^ cm^2^/s	3d, 10^−5^ cm^2^/s
H_2_O	(I)	0.98	1.36
	(II)	1.11	1.47
	(III)	-	1.81
Na^+^	(III)	-	0.84
NO_2_^−^	(III)	-	1.46

## Data Availability

All data are available from the authors upon request.
